# Auditory Verbal Hallucinations in Borderline Personality Disorder and the Efficacy of Antipsychotics: A Systematic Review

**DOI:** 10.3389/fpsyt.2018.00347

**Published:** 2018-07-31

**Authors:** Christina W. Slotema, Jan Dirk Blom, Marieke B. A. Niemantsverdriet, Iris E. C. Sommer

**Affiliations:** ^1^Department of Personality Disorders, Parnassia Psychiatric Institute, The Hague, Netherlands; ^2^Faculty of Social and Behavioural Sciences, Leiden University, Leiden, Netherlands; ^3^Department of Psychiatry, University of Groningen, Groningen, Netherlands; ^4^Department of Neuroscience, University Medical Center Groningen, Groningen, Netherlands; ^5^Department of Psychiatry, University Medical Center Utrecht, Utrecht, Netherlands

**Keywords:** prevalence, phenomenology, psychotic symptoms, childhood trauma, comorbidity

## Abstract

**Background:** Auditory verbal hallucinations (AVH) are experienced more frequently by patients with borderline personality disorder (BPD) than previously assumed. However, consensus is lacking on how to treat them.

**Objective:** To provide a systematic review of studies reporting on AVH in patients with BPD, with a focus on the efficacy of treatment of psychotic symptoms.

**Methods:** For this review a systematic search was made in the PubMed and Ovid databases, and mean weighted prevalence rates, adjusted for sample size, were computed.

**Results:** The search yielded 36 studies describing a total of 1,263 patients. Auditory hallucinations (including AVH) were reported in 27% of hospitalized BPD patients; AVH were reported in 25% of all patients and in 24% of outpatients. Of the hallucinating patients, 78% experienced AVH at least once per day, for a duration of several days to many years. On the whole, patients with BPD regarded their voices as malevolent and omnipotent in nature. Compared to patients with schizophrenia, the phenomenological characteristics of AVH were similar and the ensuing distress was equal or even higher, whereas scores for other positive symptoms were lower. The presence of AVH in BPD was associated with an increase of suicide plans and attempts, and more frequent hospitalization. Moreover, AVH in the context of BPD were associated with higher prevalence rates for post-traumatic stress disorder and emotional abuse. The efficacy of antipsychotics was investigated in 21 studies. Based on these studies, we conclude that both typical and atypical antipsychotics tend to have positive effects on AVH experienced in the context of BPD. The efficacy of cognitive-behavioral therapy and non-invasive brain stimulation has not yet been systematically assessed.

**Conclusions:** These findings indicate that AVH experienced in the context of BPD are in need of proper diagnosis and treatment, and that antipsychotics tend to be beneficial in treating these (and other psychotic) symptoms.There is an urgent need for studies assessing the efficacy of cognitive-behavioral therapy and non-invasive brain stimulation in this underdiagnosed and undertreated group.

## Introduction

Auditory verbal hallucinations (AVH) are voices perceived during wakefulness, in the absence of an external origin (9). The highest prevalence of these phenomena is in patients diagnosed with schizophrenia [i.e., 70–80%, (10)]. In these patients they tend to also yield high levels of distress which may lead to destructive behavior, such as suicide and homicide (11, 12). Although less well-known, AVH are experienced in the context of borderline personality disorder (BPD) as well (10, 13, 14). BPD is a severe psychiatric disorder with a lifetime prevalence rate of 1–3% in the general population (15, 16). It is conceptualized as an emotion-regulation disorder (17), characterized by unstable relationships, identity disturbances, and self-destructive behavior. Comorbidity with depression and other psychiatric disorders is high (18). AVH experienced by patients with BPD have long been characterized as “pseudohallucinations,” since they were thought to be mild and phenomenologically different from those in schizophrenia (19). This line of thought is still reflected in the operational criteria for BPD featuring in the fifth edition of the *Diagnostic and Statistical Manual of Mental Disorders* [DSM-5; (20)], which states that, “*Some individuals develop psychotic-like symptoms (e.g., hallucinations, body-image distortions, ideas of reference, hypnagogic phenomena) during times of stress*.” However, valid definitions for terms like “pseudohallucination” and “psychotic-like” symptoms are lacking (9).

Furthermore, there are indications that AVH are frequent and severe in the context of BPD, and that their phenomenological characteristics do not differ essentially from those of AVH in patients diagnosed with schizophrenia (2). Therefore, we prefer to regard AVH in patients with BPD as genuine hallucinations.

### Rationale

Because of the burden caused by AVH in the context of BPD, and the ensuing risk of hospitalization and an unfavorable outcome, there is an urgent need for effective treatment of these perceptual phenomena (21, 22).

### Objective

Since therapeutic guidelines for this specific purpose are lacking, this study aims to present a systematic review of the literature on AVH in BPD, with special focus on the efficacy of treatment for psychotic symptoms in this patient group. Therefore, the aim of this study was to answer the following questions in patients with BPD:

1. What is the prevalence of AVH?
2. What are the phenomenological characteristics and severity of AVH?
3. What is the association between AVH and comorbid disorders and childhood trauma?
4. What is the efficacy of treatment of psychotic symptoms?

## Methods

### Study design

A systematic review was conducted of studies reporting on the treatment of AVH and other psychotic symptoms in the context of BPD.

### Search strategy

A systematic search was performed using the PubMed and Ovid databases from 1806 through April 2017 (Medline 1946-, Embase 1974-, PsycINFO 1806-, Cochrane DSR, ACP, Journal Club, DARE, CCTR, CMR, HTA, NHSEED).

The following search terms were used: hallucination, auditory hallucination, auditory verbal hallucination, delusions, hallucinations, acute psychosis, childhood psychosis, hallucinosis, “paranoia (psychosis),” schizophrenia, affective psychosis/ or alcoholic psychosis/ or reactive psychosis/ or toxic psychoses, borderline state, “fragmentation (schizophrenia),” psychoticism, schizophreniform disorder, schizotypy,” positive and negative symptoms,” paranoid schizophrenia, undifferentiated schizophrenia, antipsychotic (drugs/agents), neuroleptic (drugs/agents), cognitive-behavioral therapy, behavior therapy, cognitive therapy, brain stimulation, direct-current stimulation, transcranial magnetic stimulation, clinical study, and borderline personality disorder.

### Data sources, studies selections, and data extraction

Articles were considered eligible for inclusion when they were written in English and presented original data *either* regarding auditory (verbal) hallucinations in patients with a primary diagnosis of BPD, *or* regarding the efficacy of antipsychotics, cognitive-behavioral therapy (CBT), non-invasive brain stimulation or the effects on treatment on the severity of (specific) psychotic symptoms in patients with BPD. The rationale to explore these interventions is that the first treatment option for hallucinations in schizophrenia spectrum disorders is antipsychotic medication, which can induce a rapid decrease in severity (23). CBT can be applied as an augmentation to antipsychotic medication, thereby reducing anxiety and distress. Transcranial magnetic stimulation (TMS) is an experimental treatment method aimed at reducing the frequency and severity of auditory hallucinations.

Cross references were systematically assessed and checked via *Google Scholar*.

### Data analysis

Mean weighted prevalence rates were computed using SPSS version 23.0. The total number of patients with AVH was divided by the total number of patients who participated into the studies presenting prevalence rates.

The severity of other psychotic symptoms was explored in a population of 107 outpatients with BPD with and without hallucinations (14). In that study, the presence of hallucinations was associated with the presence of delusions, but not with negative symptoms and disorganization. For the present review, additional analyses were performed to investigate comorbid psychotic symptoms in patients with BPD with and without AVH (*n* = 101). For this, patients were divided into two groups: (i) patients with AVH occurring at least weekly, and (ii) patients with less frequent AVH or no AVH at all.

To investigate an association between AVH and comorbid disorders and childhood trauma, we repeated the analyses (in which associations between hallucinations and the number of comorbid psychiatric disorders, and PTSD and emotional abuse were established) of the study by Niemantsverdriet et al. (14) for BPD patients with and without AVH. For this, a logistic regression with backward selection was used to examine the association between AVH and specific comorbid psychiatric disorders. These disorders were clustered into three groups, i.e., mood disorders (unipolar depression, bipolar I and II disorder), post-traumatic stress disorder (PTSD), and substance use disorders (i.e., alcohol abuse and dependence, drug abuse and dependence, or both).

Furthermore, we used a proportional odds model with backward selection to study the association between AVH and five subtypes of childhood trauma.

## Results

**Figure 1:**
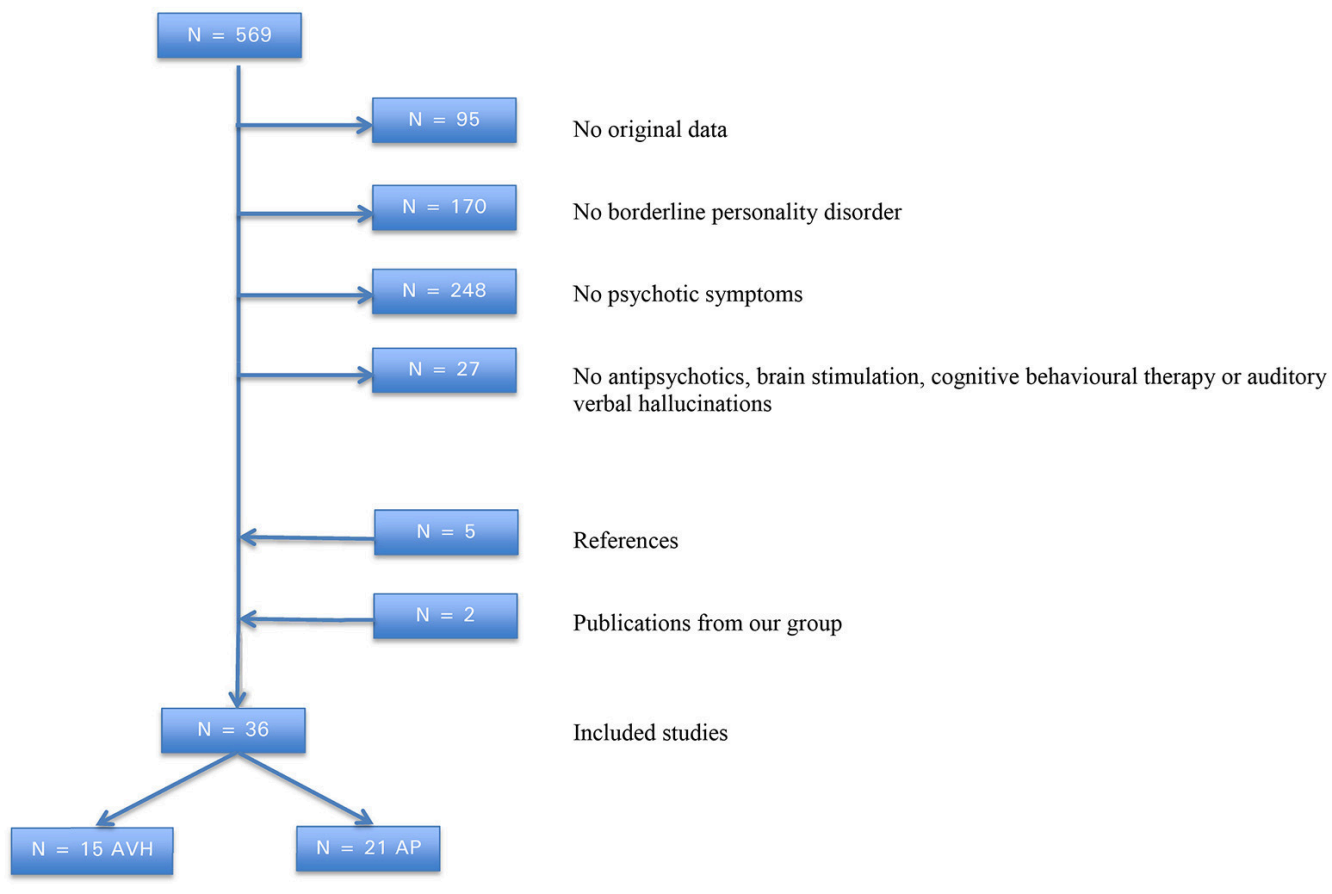
Flowchart of literature search on borderline personality disorder, auditory verbal hallucinations and antipsychotics.

The literature search yielded 569 publications on AVH and/or the use of antipsychotics in patients with BPD. Of these, 36 studies (with a total of 1,263 patients) were potentially eligible for this systematic review. Thirty-six studies were considered eligible for inclusion: 15 on AVH (with a total of 677 patients) and 22 on the treatment of psychotic features. One study was included in both systematic reviews. Of the latter 22 studies, 21 assessed the efficacy of antipsychotic medications in a total of 572 patients. Reasons for exclusion are described in Figure 1. An overview of the included studies is presented in Table 1, 2. In one study, the efficacy of combined early intervention for BPD and specialized first-episode psychosis treatment was explored (54).

**Table 1:** Studies included in the present review (*n* = 15).

**Study**	**(*n*)**	**In/outpatients**	**Diagnostic instrument**	**AVH instrument**	**Other instruments**	**Prevalence A(V)H**	**Comments**
Lotterman (24)	8 females	Inpatients	–	–	–	NA	Case report
Chopra and Beatson (25)	13 (85% female)	Inpatients	DIBP	DIBP	–	Point prevalence AH 54%	
George and Soloff (26)	24 (77% female)	Inpatients	DIB	SSI, Meehl scale	–	AH 21%	
Suzuki et al. (27)	5 females	Inpatients	DSM IV	–	–	NA	Case report
Yee et al. (19)	171 (90% female)	Outpatients	DIB-R	SCL-90	DES, SCID-D, McGuffin's Opcrit Questionnaire	Point prevalence 29%	
Barnow et al. (28)	1 female	Inpatient	–	–	–	NA	Case report
Kingdon et al. (29)	33 (76% female)	In- and outpatients	SCID II	PSYRATS	CTQ	Point prevalence 46%	Versus schizophrenia
Adams and Sanders (30)	7 (86% female)	Outpatients	–	PSYRATS, PSE	–	NA	Case report
Slotema et al. (2)	38 females	Outpatients	SCID II	PSYRATS	–	NA	Versus schizophrenia and individuals without psychiatric diagnoses
Hepworth et al. (31)	22 (60% female)	In- and outpatients	SCID II	PSYRATS	SCID I, BAVQ	NA	Versus schizophrenia Overlap with (29)
Pearse et al. (32)	30 (90% female)	Outpatients	DSM IV	PSE	–	Lifetime prevalence 50%	
Tschoeke et al. (33)	23 females	Inpatients	DSM IV-TR	PANSS	NA	NA	Versus schizophrenia patients
Slotema et al. (22)	89 (92% female)	Outpatients	SCID II	PSYRATS	MINI plus, hospitalization	See Niemantsverdriet et al. (14)	Overlap with (14)
Niemantsverdriet et al. (14)	324, subgroup 107 patients (93% female)	Outpatients	DSM IV-TR	Tailor-made interview by asking if the patient ever heard/saw/tasted/ smelled or felt something that other people did not perceive, or for which they had no explanation, including frequency and content, subgroup: PANSS, PSYRATS	MINI Plus, CTQ	Point prevalence AH 27%, AVH 21%	
Slotema et al. (21)	38 females	Outpatients	SCID II	PSYRATS	BAVQ, SCRS, VPDS	NA	Overlap with (14)

*AH, auditory hallucinations; AVH, auditory verbal hallucinations; BAVQ, Beliefs About Voices Questionnaire; CTQ, Childhood Trauma Questionnaire; DES, Dissociative Experience Scale; DIB-R, Diagnostic Interview for Borderlines Revised; DIBP, Diagnostic Interview for Borderline Patients; MINI Plus, Mini International Neuropsychiatric Interview Plus; NA, not applicable; PANSS, Positive And Negative Syndrome Scale; PSE, Present State Examination; PSYRATS, Psychotic Symptom Rating Scales; SCID II, Structured Clinical Interview for DSM IV axis 2 Personality Disorders; SCID-D, Structured Clinical Interview for DSM IV dissociative disorders; SCL-90, Symptom Check List 90 items; SCRS, Social Comparison Rating Scale; SSI, Schizotypal Symptom Inventory; VPDS, Voice Power Differential Scale*.

**Table 2:** Borderline personality disorder (BPD) and antipsychotics for severity of psychotic symptoms.

**Study**	**Design**	**Active (*n*)**	**Placebo/ control (*n*)**	**Antipsychotic**	**Dosage mean equivalent haloperidol**	**Instrument**	**Effect**	**Notification**
Serban and Siegel (34)	RCT	26	26	Thiothixene vs. haloperidol	2.8 mg 3 mg	PAI Paranoid ideation	+	BPD 31%, SPD 27% or mixed 31% thiothixene superior to haloperidol Mild transient psychotic episode before admission
Goldberg et al. (35)	RCT placebo	24	26	Thiothixene 12 weeks	2.6 mg	HSCL-90 Anger-hostility psychotic SIB Ideas of reference Paranoid ideation Delusions/hallucinations Psychotic symptoms	– + – – –	BPD 34%, SPD 26% or mixed 40% and at least one psychotic symptom
Soloff et al. (36)	RCT placebo	28	28	Haloperidol 6 weeks	4.8 mg	SCL-90 Paranoid ideation	+	BPD 39%, SPD 4%, or mixed 57%, vs. amitriptyline vs. placebo
Soloff et al. (37)	RCT placebo	36	34	Haloperidol 5 weeks	3.9 mg	SCL-90 Paranoid ideation IMPS Paranoid ideation	– –	BPD with or without SPD vs. phenelzine
Zanarini and Frankenburg (38)	RCT placebo	19	9	Olanzapine 6 months	4 mg	SCL-90 Paranoid ideation	+	
Nickel et al. (39)	RCT placebo	26	26	Aripiprazole 8 weeks	7.5 mg	SCL-90 Paranoid ideation	+	
Pascual et al. (40)	RCT placebo	30	30	Ziprasidone 12 weeks	5.3 mg	BPRS Psychotic symptoms Hallucinations CGI-BPD Paranoid ideation	– – – –	
Shafti and Shahveisi (41)	RCT	14	14	Haloperidol vs olanzapine 8 weeks	7.1 mg 5.1 mg	BPRS Psychotic symptoms Paranoid ideation	– +	
Shafti and Kaviani (42)	RCT	12	12	Olanzapine vs aripiprazole 8 weeks	4.8mg 3.5 mg	BPRS Paranoid ideation	+	Significant for aripiprazole, not for olanzapine
Teicher et al. (43)	Open label	12		Thioridazine 12 weeks	2.3 mg	SCL-90 Paranoid ideation	–	
Frankenburg and Zanarini (44)	Cases rerated	15		Clozapine 2-9 months	6.3 mg	BPRS Psychotic symptoms Paranoid ideation Hallucinations	+ + +	Treatment-resistant psychotic patients, 47% also SPD, all patients severe abuse during childhood
Suzuki et al. (27)	Case series	2		Antipsychotic deposit injection haloperidol	9-18 mg	Auditory (verbal) hallucinations Delusions and hallucinations	+ +	
Benedetti et al. (45)	Open label	12		Clozapine 16 weeks	1.1 mg	BPRS Psychotic symptoms	+	With severe psychotic-like symptoms
Schulz et al. (46)	Open label	11		Olanzapine 8 weeks	5.8 mg	BPRS Psychotic symptoms SCL-90 Paranoid ideation	+ +	
Rocca et al. (47)	Open label	15		Risperidone 8 weeks	5 mg	BPRS Paranoid ideation	+	
Pascual et al. (48)	Open label	12		Ziprasidone 2 weeks	6.4 mg	BPRS Psychotic symptoms SCL-90-R Paranoid ideation	+ +	
Mobascher et al. (49)	Cases	3		Aripiprazole up to 9 weeks	5-12.5 mg	Psychotic symptoms	+	
Perella et al. (50)	Open label	29		Quetiapine 12 weeks	10.1 mg	BPRS Paranoid ideation	+	
Mauri et al. (51)	Naturalistic study	13		Quetiapine 2 weeks	10.4 mg	PANSS positive items	+	Compared to patients with schizophrenia and drug-induced psychosis
Anjee et al. (52)	Open label	16		Quetiapine 8 weeks	10.1 mg	SCL-90 Paranoid ideation SIB Ideas of reference Paranoid ideation Delusions/hallucinations	+ + + +	
Martin-Bianco et al. (53)	Open label	12		Asenapine 8 weeks	5.8 mg	CGI-BPD Paranoid ideation	−	

*BPRS, Brief Psychiatric Rating Scale; CGI-BPD, Clinical Global Impression for Borderline Personality Disorder; HSCL-90, Hopkins Symptom Checklist 90; IMPS, Inpatients Multidimensional Psychiatric Scale; PANSS pos, Positive And Negative Syndrome Scale positive items; RCT, randomised controlled trial; SCL-90, Symptom Checklist 90; SIB, Schedule for Interviewing Borderlines; SIB, Schedule for Interviewing Borderlines; SPD, schizotypal personality disorder*.

### Prevalence rates of AVH

Of the 15 studies on AVH, six studies reported the prevalence rates for auditory hallucinations and AVH in 21–46% of the patients, the mean weighted point prevalence of AVH was 25% (14, 19, 29). With the exception of some patients in the study of Kingdon et al. (29), all study populations consisted of *outpatients*. For outpatients, the mean weighted point prevalence of AVH was 24%. In two patient samples of up to 37 *hospitalized* patients, the prevalence of auditory hallucinations ranged from 21 to 38% (25, 26) with a mean weighted prevalence of **27%**. The lifetime prevalence for auditory hallucinations was **50%** (32).

### Phenomenology and severity of AVH

In the studies in this review, the mean frequency of AVH in BPD ranged from once per day to once per hour, with each episode lasting several minutes or more (2, 14, 29). The mean onset of AVH was reported to be up to 18 years previously (2, 14, 19, 32). About half of the patients reported more than one voice (19, 32), speaking in the second person and, sometimes, in the third person (32). These voices were experienced as “comments” by 61% of the patients and as “dialogues” by 40% (33); in addition, 56% experienced imperative hallucinations (21). Control over the voices was virtually absent (2, 14, 19, 29). In four case reports (describing seven outpatients and nine inpatients), 75% were criticized by their voices, whereas commands (e.g., to hurt themselves) were perceived by 50% (24, 27, 28, 30). The duration of experiencing episodes with AVH ranged from several days to many years (24, 27, 28, 30). Voices are present, in particular, during times of stress (24, 27, 30). Compared to individuals with AVH without a psychiatric disorder, patients with BPD and AVH tend to show much higher scores on the majority of items on AVH questionnaires [e.g., “frequency,” “duration,” and “controllability”; (2)]. Regarding the phenomenological characteristics of AVH in BPD, no differences were found compared to those reported in schizophrenia (2, 29, 32, 33)].

The content of voices in BPD tends to be negative and critical (2, 14, 19, 29). Compared to patients with schizophrenia, the ensuing distress was equal to or even higher in patients with BPD (2, 29). This is in marked contrast with AVH experienced by individuals without a psychiatric diagnosis, which tend to be less frequent, mild and often benevolent (2). Two studies explored beliefs about voices (21, 31). In 10 patients with BPD, in 23 with schizophrenia and in 12 patients with both disorders, no substantial differences were found regarding beliefs about the power and malevolent intent of the participants' dominant voices (31); in all groups, the scores on these items were high. The majority of a sample of 38 patients with BPD with AVH rated their voices as malevolent and omnipotent, as well as higher in social rank than themselves (21).

The risks for suicidal attempts and hospitalization, associated with AVH, were explored in a sample of 27 outpatients with BPD and AVH, and in 62 without AVH (22). The presence of AVH was associated with a significantly higher incidence of suicidal plans and attempts in the month prior to study participation, a greater number of hospitalizations, and a shorter interval until hospitalization. In the latter study, the Psychotic Symptom Rating Scales (PSYRATS) was used as an outcome measure (55). All subscales of the PSYRATS showed a positive correlation with suicide plans. The phenomenological subscale and the subscale for distress due to AVH also showed a positive correlation with suicide attempts. Moreover, higher scores for distress due to AVH were associated with more frequent hospital admissions.

The severity of other psychotic symptoms was explored in a population of 107 outpatients with BPD with and without hallucinations (14). For the present review, additional analyses were performed to investigate comorbid psychotic symptoms in patients with and without AVH (*n* = 101). Of the 28 hallucinating patients, 78% experienced AVH at least once per day, 79% experienced at least one hallucination in a different sensory modality (i.e., visual, tactile, olfactory or gustatory), and 61% experienced additional hallucinations in multiple sensory modalities. Among patients with AVH, delusions were present in 44% of the cases, hallucinatory behavior in 70%, and suspiciousness and/or ideas of persecution in 33%.

In another study, the severity of positive symptoms in patients with BPD who experienced AVH was compared to that in patients with schizophrenia and AVH (33). In the year prior to study inclusion, in 23 patients with schizophrenia and in 21 patients with BPD, the scores for most items on the Positive and Negative Syndrome Scale (56), i.e., delusions, conceptual disorganization, grandiosity, and negative symptoms, were higher for the schizophrenia group. Scores for BPD were the same as found in the present study.

In a case series of 10 patients with BPD, Yee et al. (19) explored the co-occurrence of hallucinations in various sensory modalities. In their group, 30% experienced visual and olfactory hallucinations in addition to their AVH, while all of them (100%) reported thought insertion, 90% thought blocking or withdrawal, and 70% the sensation of being under the influence of some external power. In four other case reports (describing a total of 16 patients), AVH were regularly accompanied by visual, olfactory, and tactile hallucinations, as well as by paranoid delusions. In addition, one patient experienced ideas of reference (24, 27, 28, 30).

### Comorbid disorders and childhood trauma

Very few studies have investigated the association between AVH and comorbid psychiatric disorders. Tschoeke et al. (33) explored childhood abuse and dissociative disorders in 23 BPD patients with AVH, and in 21 patients with schizophrenia. The BPD patients more frequently reported childhood trauma, notably emotional abuse and neglect. Moreover, the BPD patients scored higher than the schizophrenia patients on scales for emotional and physical abuse and neglect, and on scales for sexual abuse. Major dissociative disorders were present in 96% of the BPD patients (78% dissociative disorder not otherwise specified, and 18% dissociative identity disorder), whereas they were absent in patients diagnosed with schizophrenia. We repeated the analyses of the study by Niemantsverdriet et al. (14) for BPD patients with and without AVH. A best fit was achieved by including PTSD and excluding mood disorders and substance use disorders. For patients with PTSD, the odds ratio (OR) for experiencing AVH, as compared to patients without PTSD, was 2.7 (CI 1.2–7.2, *p* = 0.021). No significant associations were found between the presence of AVH and comorbid mood or substance-use disorders. In addition, our analyses showed that the median number of comorbid diagnoses was higher for BPD patient with AVH than for those without (four vs. three, respectively; Kendall's tau-b 0.199, *p* = 0.021). In addition, a best fit was achieved for emotional abuse by dropping the variables for the other four subtypes, i.e., sexual abuse, physical abuse, and physical, and emotional neglect. The OR for emotional abuse was 1.1 (CI 1.014–1.220, *p* = 0.024).

### Treatment of psychotic symptoms in patients with BPD

Our search yielded 21 studies on treatment with antipsychotics in BPD, describing a total of 572 patients (Table 2).

We found no comparable studies in the field of CBT or non-invasive brain stimulation, i.e., transcranial direct current stimulation (tDCS) and TMS. However, Gleeson et al. (54) reported on a randomized controlled pilot study with 16 patients (aged 15–25 years) in which they compared combined specialist first-episode treatment plus specialist early intervention for borderline personality, entitled Helping Young People Early (HYPE; using an integrated, team-based intervention model comprising time-limited cognitive-analytic therapy, case management, and general psychiatric care) with specialist first-episode treatment (SFET) alone (2012). Their aim was to provide treatment for both psychosis and borderline personality. Although the numbers were too small to allow for significance testing or the calculation of effect sizes, the HYPE + SFET group showed fewer positive psychotic and negative symptoms, less severe anhedonia and depression, better functioning, and better adherence to medication than the SFET group. However, a larger randomized controlled trial (RCT) is needed to confirm these results.

In four studies describing antipsychotic treatment for patients with BPD *and* psychotic features/disorders (34, 35, 44, 45), one included only treatment-resistant psychotic patients with BPD, which were subsequently recruited for a larger study on the efficacy of clozapine for treatment-resistant psychotic patients (44). In three of the four studies, the severity of psychotic phenomena decreased. The other studies included patients with BPD without distinctions for psychotic features. Of the 21 studies investigating the efficacy of antipsychotic medication, nine were RCTs with 215 patients in the active condition and 205 in the placebo condition (34–42). In three of these studies the severity of psychotic symptoms was explored, but no decrease in severity was found in any of them. Paranoid symptoms diminished in six out of nine studies; the remaining 12 were open-label, naturalistic, or case studies, with a total of 152 patients. After treatment with antipsychotic medication, the severity of psychotic symptoms was reduced in all six studies that assessed this, and the severity of paranoid symptoms was reduced in six of eight studies. The severity of hallucinations was included as an outcome measure in only one study (44); in that study clozapine reduced the severity of therapy-resistant AVH. No clinical trials were found in which the severity of AVH was used as an outcome measure. However, AVH subsided after treatment with antipsychotics in two patients described in a case report (27). The antipsychotic dosages used in seven studies were 1,3 up to 5 times lower compared to those regularly used for patients with psychotic disorders (34, 35, 37, 38, 42, 43, 45), while the other studies applied regular dosages. However, antipsychotics were effective in reducing psychotic symptoms, even at low dosages. In one study, the effect of quetiapine was explored in patients with BPD, drug-induced psychosis, and schizophrenia (51). At the end of that study, the mean scores of each rating scale were significantly lower in the population as a whole, and also within each diagnostic group. The percentage improvement was significantly greater in patients with drug-induced psychosis than in those with schizophrenia or BPD. The mean weighted percentage of dropouts in patients using typical antipsychotics was 36 and 43% for atypical antipsychotics. Among the atypical antipsychotics, side effects occurred with the following mean weighted percentages: 42% for sedation, 78% for drooling, 44% for weight gain, 29% for nausea, 22% for dizziness, 11% for tremor, 11% for anxiety, 18% for irritability, 63% for increased appetite, 38% for dry mouth, 15% for akathisia, 13% for confusion, 14% for insomnia, 11% for headache, and 5% for other side effects. The mean weight gain associated with the use of olanzapine ranged from 1.3 to 4.5 kg. The side effects of the typical antipsychotic thioridazine (the only publication of typical antipsychotics reporting side effects) were dry mouth (58%), dizziness (50%), sedation (42%), weight gain (33%), constipation 17%, and akathisia (8%).

## Discussion

This systematic review presents an update on auditory verbal hallucinations (AVH) among patients with borderline personality disorder (BPD), with a special focus on the efficacy of treatments for psychotic symptoms. It was found that AVH in BPD are generally severe and in need of effective treatment. Auditory hallucinations, including AVH, were reported in 27% of inpatients with BPD. AVH were experienced in 25% of all patients and in 24% of the outpatients. These results are positioned between the prevalence rates for AVH in the general population, i.e., 9% (4, 7), and in schizophrenia [70–80%, (10)]. Of all patients with BPD and AVH, 78% perceived their AVH at least once per day and the ensuing distress was rated as high. The duration of experiencing AVH ranged from several days to many years. These findings are in contrast with the older literature which characterizes AVH in the context of BPD as mild and transient (20, 57). Furthermore, the phenomenological characteristics of AVH in patients with BPD do not differ from those in schizophrenia, and the ensuing distress might be even higher. Patients with BPD tend to experience their voices as malevolent and omnipotent, and higher in social rank than themselves. These findings are in line with those for schizophrenia (31, 58). The majority of BPD patients with AVH experienced hallucinations in at least one other sensory modality, with visual hallucinations being the most prominent. Scores for delusions were positive in 44% of the patients, and for suspiciousness and ideas of persecution in 33%. However, compared to patients diagnosed with schizophrenia, the overall scores for positive and negative symptoms were lower in patients with BPD (10, 33).

The prevalence rate for imperative hallucinations of 56% is in line with the findings that the presence of AVH in the context of BPD was associated with an increase in the incidence of suicidal plans and attempts, as well as with the number of hospitalizations. The same applies for suicidality in patients with AVH in schizophrenia spectrum disorders (59, 60). Comorbidity (e.g., with mood disorders and substance use disorders) has sometimes been held responsible for the occurrence of AVH in patients with BPD (3). In two studies the relationship of AVH with comorbidity and childhood trauma was investigated. In the present BPD group, PTSD was the only comorbid disorder associated with AVH. Comorbid dissociative disorders were more prevalent among patients with AVH and BPD compared to those with schizophrenia (33). The number of patients with childhood abuse was higher in BPD patients with AVH than in those without AVH. These findings are similar to those for childhood abuse reported for psychotic disorders (61).

Psychotherapies, notably dialectical behavior therapy, are effective for BPD core pathology and associated general psychopathology (62, 63). Of the 22 studies that assessed the efficacy of treatments for psychotic features in patients with BPD, only one assessed the efficacy of a first-episode specialty program, and none evaluated the efficacy of CBT, tDCS or rTMS for AVH. In most studies the severity of psychotic symptoms and paranoid delusions decreased with the use of antipsychotics; however, there were considerable dropouts and side effects (the most common being drooling, increased appetite, sedation, dry mouth, and weight gain).

Although the number of studies was low, these findings indicate that antipsychotics tend to have a beneficial effect on psychotic symptoms experienced in the context of BPD. Moreover, it should be noted that, since the majority of studies (i.e., 17 of 21) included patients with *and* without psychotic symptoms, the antipsychotics would probably have shown greater benefit if patients with psychotic symptoms *alone* would have been included. Furthermore, the effects of antipsychotics are expected to increase with higher dosages. These results are in line with other meta-analyses which found small to moderate effect sizes for antipsychotics in the treatment of psychotic symptoms in patients with BPD (64–66). In one study, the effects of antipsychotics did not differ from those in patients with schizophrenia (51). Finally, patients with BPD and psychotic disorders might benefit from a combination of a specialist first-episode treatment plus specialist early intervention for borderline personality, as investigated by Gleeson et al. (54).

### Implications for clinical practice and future research

The findings presented in this systematic review have four main implications. Firstly, AVH among patients with BPD are common. Since they are associated with high levels of distress and their consequences can be severe, these patients deserve appropriate treatment for their AVH. This is in contrast with the older literature, where psychotic symptoms are sometimes dismissed as “psychotic-like” symptoms or “pseudohallucinations” and, therefore, do not get the attention they deserve (30). Secondly, our findings indicate that the psychotic symptoms experienced by patients with BPD resemble those experienced by patients diagnosed with schizophrenia (although delusions, negative symptoms and disorganization are less common). Therefore, AVH as experienced in the context of BPD appear to lie on a continuum with those in individuals without a psychiatric diagnosis, and those with schizophrenia. This is in line with the original meaning of the term “borderline disorder” which was introduced to designate a condition in the borderline area between neurotic and psychotic disorders, with the possibility of occasional transgressions of the border in either direction (67). Although even BPD patients with persistent hallucinations are rarely diagnosed with a DSM-5 diagnosis of schizophrenia spectrum disorder (30), we argue that, in specific cases, it would be better to diagnose both disorders and offer treatment accordingly, especially when the distress caused by those hallucinations is high. Thirdly, our findings also imply that patients with BPD and psychotic symptoms are likely to benefit from antipsychotic medication. When side effects are mild or absent, there is no reason to withhold these patients from higher dosages if lower dosages fail to yield adequate effects. Fourth and finally, patients with BPD and AVH might benefit from other treatments for psychotic disorders, such as CBT and non-invasive brain-stimulation techniques (1, 5). In CBT, control over symptoms and problems can be obtained with the aid of a personalized case formulation. Of special interest is the frequent presence of command hallucinations in BPD, which may lead to suicidal behavior (68). These patients might benefit from CBT specifically designed for the treatment of command hallucinations (69). rTMS and tDCS are thought to normalize increased brain activity with a rapidly changing magnetic field, and a low-intensity current, respectively. Patients undergo these treatment under full consciousness. If applied in conformance with international safety guidelines. The modalities rTMS and tDCS are safe and their side effects (if present) are temporary and mild. Moreover, non-invasive brain stimulation is beneficial in patients with psychotic features and AVH (6, 8, 70–72).

### Limitations

An important limitation of the present study is the relatively low number of original studies and numbers of patients included per study. Moreover, the use of differing assessment methods and questionnaires in the original studies makes it difficult to directly compare their findings, and is therefore a minor limitation.

## Conclusions

AVH have a mean weighted prevalence of 26% in patients with BPD. They tend to be experienced as severe and their phenomenological characteristics do not differ from those in patients diagnosed with schizophrenia. These findings emphasis the need for proper diagnosis and appropriate treatment. There is some evidence that antipsychotics are effective in the treatment of psychotic symptoms in patients with BPD. In addition, these patients might benefit from other treatments for psychosis, such as CBT and non-invasive brain stimulation. Future studies should be directed at assessing the effects of such treatment strategies for this underserved subpopulation.

## Author contributions

CS contributed to the conception and design of the work, and to the acquisition, analysis, and interpretation of data for the work, drafted and revised the work, gave final approval for the final version to be published, and agreed to be accountable for all aspects of the work in ensuring that questions related to the accuracy or integrity of any part of the work are appropriately investigated and resolved. JB and IS contributed to the conception and design of the work, and to the analysis and interpretation of data for the work, revised the work, gave final approval for the final version to be published, and agreed to be accountable for all aspects of the work in ensuring that questions related to the accuracy or integrity of any part of the work are appropriately investigated and resolved. MN contributed to the conception and design of the work, to the analysis and interpretation of data for the work, drafted and revised the work, gave final approval for the final version to be published, and agreed to be accountable for all aspects of the work in ensuring that questions related to the accuracy or integrity of any part of the work are appropriately investigated and resolved.

### Conflict of interest statement

The authors declare that the research was conducted in the absence of any commercial or financial relationships that could be construed as a potential conflict of interest.

## References

[1] Slotema CW Aleman A Daskalakis ZJ Sommer IEC. Meta-analysis of repetitive transcranial magnetic stimulation in the treatment of auditory verbal hallucinations: update and effects after one month. Schizophr Res. (2012) 142:40–5. 10.1016/j.schres.2012.08.025 23031191

[2] Slotema CW Daalman K Blom JD Diederen KM Hoek HW Sommer IEC. Auditory verbal hallucinations in patients with borderline personality disorder are similar to those in schizophrenia. Psychol Med. (2012) 42:1873–8. 10.1017/S0033291712000165 22336487

[3] Schroeder K Fisher HL Schäfer I. Psychotic symptoms in patients with borderline personality disorder: prevalence and clinical management. Curr Opin Psychiatry (2013) 26:113–9. 10.1097/YCO.0b013e32835a2ae7 23168909

[4] Beavan V Read J Cartwright C. The prevalence of voice-hearers in the general population: a literature review. J Ment Health (2011) 20:281–92. 10.3109/09638237.2011.562262 21574793

[5] Burns AM Erickson DH Brenner CA. Cognitive-behavioral therapy for medication-resistant psychosis: a meta-analytic review. Psychiatr Serv. (2014) 65:874–80. 10.1176/appi.ps.201300213 24686725

[6] Koops S van den Brink H Sommer IEC. Transcranial direct current stimulation as a treatment for auditory hallucinations. Front Psychol. (2015) 6:244. 10.3389/fpsyg.2015.00244 25798123 PMC4351567

[7] Maijer K Begemann MJH Palmen SJMC Leucht S Sommer IEC Auditory hallucinations across the lifespan: a systematic review and meta-analysis. Psychol Med. (2017) 28:1–10. 10.1017/S0033291717002367 28956518

[8] Otani VH Shiozawa P Cordeiro Q Uchida RR. A systematic review and meta-analysis of the use of repetitive transcranial magnetic stimulation for auditory hallucinations treatment in refractory schizophrenic patients. Int J Psychiatry Clin Pract. (2015) 19:228–32. 10.3109/13651501.2014.980830 25356661

[9] Blom JD A Dictionary of Hallucinations. New York, NY: Springer (2010).

[10] Larøi F Sommer IEC Blom JD Fernyhough C Ffytche DH Hugdahl K The characteristic features of auditory verbal hallucinations in clinical and non-clinical groups: state-of-the-art overview and future directions. Schizophr Bull. (2012) 38:724–33. 10.1093/schbul/sbs061 22499783 PMC3406519

[11] Cheung P Schweitzer I Crowley K Tuckwell V. Violence in schizophrenia: role of hallucinations and delusions. Schizophr Res. (1997) 26:181–90. 10.1016/S0920-9964(97)00049-2 9323349

[12] Wong M Fenwick P Fenton G Lumsden J Maisey M Stevens J. Repetitive and non-repetitive violent offending behaviour in male patients in a maximum security mental hospital—clinical and neuroimaging findings. Med Sci Law (1997) 37:150–60. 10.1177/002580249703700211 9149510

[13] Lindley SE Carlson E Sheikh J. Psychotic symptoms in posttraumatic stress disorder. CNS Spectr. (2000) 5:52–7. 10.1017/S1092852900021659 17637580

[14] Niemantsverdriet MB Slotema CW Blom JD Franken IH Hoek HW Sommer IEC. Hallucinations in borderline personality disorder: prevalence, characteristics and associations with comorbid symptoms and disorders. Sci Rep. (2017) 7:13920. 10.1038/s41598-017-13108-6 29066713 PMC5654997

[15] Lenzenweger MF Lane MC Loranger AW Kessler RC. DSM-IV personality disorders in the National Comorbidity Survey Replication. Biol Psychiatry (2007) 62:553–64. 10.1016/j.biopsych.2006.09.019 17217923 PMC2044500

[16] Trull TJ Jahng S Tomko RL Wood PK Sher KJ. Revised NESARC personality disorder diagnoses: gender, prevalence, and comorbidity with substance dependence disorders. J Pers Dis. (2010) 24:412–26. 10.1521/pedi.2010.24.4.412 20695803 PMC3771514

[17] Crowell SE Beauchaine TP Linehan MM. A biosocial developmental model of borderline personality: elaborating and extending Linehan's theory. Psychol Bull. (2009) 135:495–510. 10.1037/a0015616 19379027 PMC2696274

[18] Grant BF Chou SP Goldstein RB Huang B Stinson FS Saha TD. Prevalence, correlates, disability, and comorbidity of DSM-IV borderline personality disorder: results from the Wave 2 National Epidemiologic Survey on Alcohol and Related Conditions. J Clin Psychiatry (2008) 69:533–45. 10.4088/JCP.v69n0404 18426259 PMC2676679

[19] Yee L Korner AJ McSwiggan S Meares RA Stevenson J. Persistent hallucinosis in borderline personality disorder. Compr Psychiatry (2005) 46:147–54. 10.1016/j.comppsych.2004.07.032 15723033

[20] American Psychiatric Association Diagnostic and Statistical Manual of Mental Disorders. 5th ed. Washington, DC: American Psychiatric Association (2013).

[21] Slotema CW Blom JD Deen M Niemantsverdriet MBA van der Gaag M Hoek HW Negative beliefs about voices in patients with borderline personality disorder predict distress and need for hospitalization. Psychopathology (2017) 50:255–61. 10.1159/000477669 28738347

[22] Slotema CW Niemantsverdriet MB Blom JD van der Gaag M Hoek HW Sommer IEC. Suicidality and hospitalisation in patients with borderline personality disorder who experience auditory verbal hallucinations. Eur Psychiatry (2017) 41:47–52. 10.1016/j.eurpsy.2016.10.003 28049081

[23] Sommer IEC Slotema CW Daskalakis ZJ Derks EM Blom JD van der Gaag M. The treatment of hallucinations in schizophrenia spectrum disorders. Schizophr Bull. (2012) 38:704–14. 10.1093/schbul/sbs034 22368234 PMC3577047

[24] Lotterman AC. Prolonged psychotic states in borderline personality disorder. Psychiatr Quart. (1985) 57:33–46. 10.1007/BF01064975 4080867

[25] Chopra HD Beatson JA. Psychotic symptoms in borderline personality disorder. Am J Psychiatry (1986) 143:1605–7. 10.1176/ajp.143.12.1605 3789216

[26] George A Soloff PH. Schizotypal symptoms in patients with borderline personality disorders. Am J Psychiatry (1986) 143:212–5. 10.1176/ajp.143.2.212 3946657

[27] Suzuki H Tsukamoto C Nakano Y Aoki S Kuroda S. Delusions and hallucinations in patients with borderline personality disorder. Psychiatr Clin Neurosci. (1998) 52:605–10. 10.1111/j.1440-1819.1998.tb02708.x 9895209

[28] Barnow S Arens EA Sieswerda S Dinu-Biringer R Spitzer C Lang S. Borderline personality disorder and psychosis: a review. Curr Psychiatry Rep. (2010) 12:186–95. 10.1007/s11920-010-0107-9 20425279

[29] Kingdon DG Ashcroft K Bhandari B Gleeson S Warikoo N Symons M Characteristics of psychosis in borderline personality disorder: similarities and differences in the experience of auditory hallucinations, paranoia, and childhood trauma. J Nerv Ment Dis. (2010) 198:399–403. 10.1097/NMD.0b013e3181e08c27 20531117

[30] Adams B Sanders T. Experiences of psychosis in borderline personality disorder: a qualitative analysis. J Ment Health (2011) 20:381–91. 10.3109/09638237.2011.577846 21770785

[31] Hepworth CR Ashcroft K Kingdon D. Auditory hallucinations: a comparison of beliefs about voices in individuals with schizophrenia and borderline personality disorder. Clin Psychol Psychother. (2013) 20:239–45. 10.1002/cpp.791 21976361

[32] Pearse LJ Dibben C Ziauddeen H Denman C McKenna PJ. A study of psychotic symptoms in borderline personality disorder. J Nerv Ment Dis. (2014) 202:368–71. 10.1097/NMD.0000000000000132 24727723

[33] Tschoeke S Steinert T Flammer E Uhlmann C. Similarities and differences in borderline personality disorder and schizophrenia with voice hearing. J Nerv Ment Dis. (2014) 202:544–9. 10.1097/NMD.0000000000000159 24921419

[34] Serban G Siegel S Response of borderline and schizotypal patients to small doses of thiothixene and haloperidol. Am J Psychiatry (1984) 11:1455–8. 10.1176/ajp.141.11.1455 6388363

[35] Goldberg SC Schulz C Schulz PM Resnick RJ Hamer RM Friedel RO. Borderline and schizotypal personality disorders treated with low-dose thiothixene vs placebo. Arch Gen Psychiatry (1986) 43:680–6. 10.1001/archpsyc.1986.01800070070009 3521531

[36] Soloff PH George A Nathan S Schulz PM Cornelius JR Herring J. Amitriptyline versus haloperidol in borderlines: final outcomes and predictors of response. J Clin Psychopharmacol. (1989) 9:238–46. 10.1097/00004714-198908000-00002 2768542

[37] Soloff PH Cornelius J George A Nathan S Perel JM Ulrich RF. Efficacy of phenelzine and haloperidol in borderline personality disorder. Arch Gen Psychiatry (1993) 50:377–85. 10.1001/archpsyc.1993.01820170055007 8489326

[38] Zanarini MC Frankenburg FR. Olanzapine treatment of female borderline personality disorder patients: a double-blind, placebo-controlled pilot study. J Clin Psychiatry (2001) 62:849–54. 10.4088/JCP.v62n1103 11775043

[39] Nickel MK Muehlbacher M Nickel C Kettler C Gil FP Bachler E. Aripiprazole in the treatment of patients with borderline personality disorder: a double-blind, placebo-controlled study. Am J Psychiatry (2006) 163:833–8. 10.1176/ajp.2006.163.5.833 16648324

[40] Pascual JC Soler J Puigdemont D Pérez-Ega R Tiana T Alvarez E. Ziprasidone in the treatment of borderline personality disorder: a double-blind, placebo-controlled, randomized study. J Clin Psychiatry (2008) 69:603–8. 10.4088/JCP.v69n0412 18251623

[41] Shafti SS Shahveisi B. Olanzapine versus haloperidol in the management of borderline personality disorder: a randomized double-blind trial. J Clin Psychopharmacol. (2010) 30:44–7. 10.1097/JCP.0b013e3181c826ff 20075647

[42] Shafti SS Kaviani H A comparative study on olanzapine and aripiprazole for symptom management in female patients with borderline personality disorder. Bull Clin Psychopharmacol. (2015) 25:38–43. 10.5455/bcp.20140923100030

[43] Teicher MH Glod CA Aaronson ST Gunter PA Schatzberg AF Cole JO. Open assessment of the safety and efficacy of thioridazine in the treatment of patients with borderline personality disorder. Psychopharmacol Bull. (1989) 25:535–49. 2631134

[44] Frankenburg FR Zanarini MC. Clozapine treatment of borderline patients: a preliminary study. Compr Psychiatry (1993) 34:402–5. 10.1016/0010-440X(93)90065-C 8131384

[45] Benedetti F Sforzini L Colombo C Maffei C Smeraldi E. Low-dose clozapine in acute and continuation treatment of severe borderline personality disorder. J Clin Psychiatry (1998) 59:103–7. 10.4088/JCP.v59n0302 9541151

[46] Schulz SC Camlin KL Berry SA Jesberger JA. Olanzapine safety and efficacy in patients with borderline personality disorder and comorbid dysthymia. Biol Psychiatry (1999) 46:1429–35. 10.1016/S0006-3223(99)00128-6 10578457

[47] Rocca P Marchiaro L Cocuzza E Bogetto F. Treatment of borderline personality disorder with risperidone. J Clin Psychiatry (2002) 63:241–4. 10.4088/JCP.v63n0311 11926724

[48] Pascual JC Oller S Soler J Barrachina J Alvarez E Pérez V. Ziprasidone in the acute treatment of borderline personality disorder in psychiatric emergency services. J Clin Psychiatry (2004) 65:1281–3. 10.4088/JCP.v65n0918b 15367057

[49] Mobascher A Mobascher J Schlemper V Winterer G Malevani J. Aripiprazole pharmacotherapy of borderline personality disorder. Pharmacopsychiatry (2006) 39:111–2. 10.1055/s-2006-941485 16721700

[50] Perella C Carrus D Costa E Schifano F Quetiapine for the treatment of borderline personality disorder: an open-label study. Progr Neuro Psychopharmacol Biol Psychiatry (2007) 31:158–63. 10.1016/j.pnpbp.2006.08.012 17045720

[51] Mauri MC Volonteri LS Fiorentini A Pirola R Bareggi SR. Two weeks' quetiapine treatment for schizophrenia, drug-induced psychosis and borderline personality disorder: a naturalistic study with drug plasma levels. Expert Opin Pharmacother. (2007) 8:2207–13. 10.1517/14656566.8.14.2207 17927477

[52] Anjee A Romine A Brown E Thuras P Lee S Schulz SC Quetiapine in patients with borderline personality disorder: an open-label trial. Ann Clin Psychiatry (2008) 20:219–26. 10.1080/10401230802467545 19034754

[53] Martin-Bianco A Patrizi B Villalta L Gasol X Soler J Gasol N Asenapine in the treatment of borderline personality disorder: an atypical antipsychotic alternative. Int Clin Psychopharmacol. (2014) 29:120–3. 10.1097/YIC.0000000000000004 23962963

[54] Gleeson JF Chanen A Cotton SM Pearce T Newman B McCutcheon L. Treating co-occurring first-episode psychosis and borderline personality: a pilot randomized controlled trial. Early Interv Psychiatry (2012) 6:21–9. 10.1111/j.1751-7893.2011.00306.x 22379625

[55] Haddock G McCarron J Tarrier N Faragher EB. Scales to measure dimensions of hallucinations and delusions: the psychotic symptom rating scales (PSYRATS). Psychol Med. (1999) 29:879–89. 10.1017/S0033291799008661 10473315

[56] Kay SR Fiszbein A Opler L. A The Positive and Negative Syndrome Scale (PANSS) for schizophrenia Schizophr Bull. (1987) 13:261–76. 10.1093/schbul/13.2.261 3616518

[57] Zanarini MC Gunderson JG Frankenburg FR. Cognitive features of borderline personality disorder. Am J Psychiatry (1990) 147:57–63. 10.1176/ajp.147.1.57 2293789

[58] Van Oosterhout B Krabbendam L Smeets G van der Gaag M Metacognitive beliefs, beliefs about voices and affective symptoms in patients with severe auditory verbal hallucinations. Br J Clin Psychol. (2013) 52:235–48. 10.1111/bjc.12011 23865402

[59] Kjelby E Sinkeviciute I Gjestad R Kroken RA Løberg EM Jørgensen HA. Suicidality in schizophrenia spectrum disorders: the relationship to hallucinations and persecutory delusions. Eur Psychiatry (2015) 30:830–6. 10.1016/j.eurpsy.2015.07.003 26443050

[60] Fuijta J Takahashi Y Nishida A Okumura Y Ando S Kawano M Auditory verbal hallucinations increase the risk for suicide attempts in adolescents with suicidal ideation. Schizophr Res. (2015) 168:209–12. 10.1016/j.schres.2015.07.028 26232867

[61] Varese F Smeets F Drukker M Lieverse R Lataster T Viechtbauer W. Childhood adversities increase the risk of psychosis: a meta-analysis of patient-control, prospective- and cross-sectional cohort studies. Schizophr Bull. (2012) 38:661–71. 10.1093/schbul/sbs050 22461484 PMC3406538

[62] Cristea IA Gentili C Cotet CD Palomba D Barbui C Cuijpers P. Efficacy of psychotherapies for borderline personality disorder: a systematic review and meta-analysis. JAMA Psychiatry (2017) 74:319–28. 10.1001/jamapsychiatry.2016.4287 28249086

[63] Stoffers JM Völlm BA Rücker G Timmer A Huband N Lieb K Psychological therapies for people with borderline personality disorder. Cochr Database Syst Rev. (2012) 8:CD005652 10.1002/146518 PMC6481907 22895952

[64] Ingenhoven TJ Duivenvoorden HJ. Differential effectiveness of antipsychotics in borderline personality disorder: meta-analyses of placebo-controlled, randomized clinical trials on symptomatic outcome domains. J Clin Psychopharmacol. (2011) 31:489–96. 10.1097/JCP.0b013e3182217a69 21694626

[65] Ingenhoven TJ Lafay P Rinne T Passchier J Duivenvoorden H. Effectiveness of pharmacotherapy for severe personality disorders: meta-analysesof randomized controlled trials. J Clin Psychiatry (2010) 71:14–25. 10.4088/JCP.08r04526gre 19778496

[66] Lieb K Völlm B Rücker G Timmer A Stoffers JM. Pharmacotherapy for borderline personality disorder: cochrane systematic review of randomised trials. Br J Psychiatry (2010) 196:4–12. 10.1192/bjp.bp.108.062984 20044651

[67] Stern A Psychoanalytic investigation and therapy in the borderline group of neuroses. Psychoanal Q. (1938) 7:467–89. 10.1080/21674086.1938.11925367

[68] Wong Z Öngür D Cohen B Ravichandran C Noam G Murphy B. Command hallucinations and clinical characteristics of suicidality in patients with psychotic spectrum disorders. Compr. Psychiatry (2013) 54:611–7. 10.1016/j.comppsych.2012.12.022 23375263

[69] Birchwood M Michail M Meaden A Tarrier N Lewis S Wykes T. Cognitive behaviour therapy to prevent harmful compliance with command hallucinations (COMMAND): a randomised controlled trial. Lancet Psychiatry (2014) 1:23–33. 10.1016/S2215-0366(14)70247-0 26360400

[70] Slotema CW Blom JD van Lutterveld R Hoek HW Sommer IEC. Review of the efficacy of transcranial magnetic stimulation for auditory verbal hallucinations. Biol Psychiatry (2014) 76:101–10. 10.1016/j.biopsych.2013.09.038 24315551

[71] Brunelin J Mondino M Gassab L Haesebaert F Gaha L Suaud-Chagny MF. Examining transcranial direct-current stimulation (tDCS) as a treatment for hallucinations in schizophrenia. Am J Psychiatry (2012) 169:719–24. 10.1176/appi.ajp.2012.11071091 22581236

[72] Pondé PH de Sena EP Camprodon JA de Araujo AN Neto MF DiBiasi M Use of transcranial direct current stimulation for the treatment of auditory verbal hallucinations of schizophrenia - a systematic review. Neuropsychiatr Dis Treat. (2017) 13:347–55. 10.2147/NDT.S122016 28203084 PMC5295799

